# Thermostable homologues of the periplasmic siderophore-binding protein CeuE from *Geobacillus stearothermophilus* and *Parageobacillus thermoglucosidasius*

**DOI:** 10.1107/S2059798323004473

**Published:** 2023-07-10

**Authors:** Elena V. Blagova, Alex H. Miller, Megan Bennett, Rosalind L. Booth, Eleanor J. Dodson, Anne-Kathrin Duhme-Klair, Keith S. Wilson

**Affiliations:** a Structural Biology Laboratory, Department of Chemistry, University of York, Heslington, York YO10 5DD, United Kingdom; b Department of Chemistry, University of York, Heslington, York YO10 5DD, United Kingdom; University of Cambridge, United Kingdom

**Keywords:** thermophilic proteins, siderophore binding, structure, biophysical characterization, CeuE, *Geobacillus stearothermophilus*, *Parageobacillus thermoglucosidasius*

## Abstract

Siderophore-binding proteins from two thermophilic bacteria, *Geobacillus stearothermophilus* and *Parageobacillus thermoglucosidasius*, were identified from a search of sequence databases, cloned and overexpressed. They are homologues of the well characterized protein *Cj*CeuE from *Campylobacter jejuni*. The iron-binding histidine and tyrosine residues are conserved in both thermophiles. Crystal structures were determined of the apo proteins and of their complexes with iron(III)-azotochelin and its analogue iron(III)-5-LICAM. The thermostability of both homologues was shown to be about 20°C higher than that of *Cj*CeuE. Similarly, the tolerance of the homologues to the organic solvent dimethylformamide (DMF) was enhanced, as reflected by the respective binding constants for these ligands measured in aqueous buffer at pH 7.5 in the absence and presence of 10% and 20% DMF. Consequently, these thermophilic homologues offer advantages in the development of artificial metalloenzymes using the CeuE family.

## Abstract 2

The expression, characterization and structures of CeuE homologues from two thermophilic bacteria, *Geobacillus stearothermophilus* and *Parageobacillus thermoglucosidasius*, are described together with their ligand binding. The proteins show enhanced thermostability and resistance to organic chemicals; consequently, these thermophilic homologues offer advantages in the development of artificial metalloenzymes using the CeuE family.

## 1. Introduction

Iron(III) is an essential nutrient that is required by most bacterial organisms for fundamental biological processes including photosynthesis, respiration, oxygen transport, iron-regulated gene expression, DNA biosynthesis *etc.* (Guerinot, 1994 ▸; Braun & Killmann, 1999 ▸; Krewulak & Vogel, 2008 ▸). Bacteria are constantly fighting for iron-dependent survival as, due to the low solubility of iron(III) in water at neutral pH, iron has low availability to bacteria (Raymond *et al.*, 2003 ▸). Pathogenic microorganisms overcome this limitation in the host by acquiring iron either extracellularly or intracellularly. This is achieved via two general mechanisms. The first is direct highly selective iron uptake based on iron acquisition from various iron sources such as lactoferrin, transferrin, ferritin, haem and/or haemoproteins (Schwiesow *et al.*, 2018 ▸; Krewulak & Vogel, 2008 ▸). This involves direct contact between the bacterium and the exogenous iron. The second mechanism is indirect siderophore-based iron acquisition, which relies on molecules (siderophores and haemophores) that are synthesized and secreted by bacteria into the extracellular medium (Wandersman & Delepelaire, 2004 ▸). The indirect strategy is capable of exploiting all available iron sources, independent of their nature, and is found among a broad spectrum of prokaryotic and eukaryotic species (Miethke, 2013 ▸; Bowden *et al.*, 2018 ▸). Under iron-deficient conditions (Baars *et al.*, 2018 ▸) bacteria have developed a successful mechanism of iron(III) uptake into the cell through the secretion of high-affinity iron-chelating molecules known as siderophores. Bacteria synthesize siderophores to capture iron(III) from the surrounding environment and transport them into the cytoplasm (Guerinot, 1994 ▸; Schwiesow *et al.*, 2018 ▸; Miethke & Marahiel, 2007 ▸; Hider & Kong, 2010 ▸; Kramer *et al.*, 2020 ▸; Marchetti *et al.*, 2020 ▸).

In Gram-negative bacteria, periplasmic binding proteins (PBPs) receive the siderophore-chelated iron from an outer membrane cell-surface receptor protein (Krewulak & Vogel, 2008 ▸; Hider & Kong, 2010 ▸) and sequester the siderophore–iron(III) complex in the periplasm, where it interacts with an inner membrane ATPase-permease/ABC transporter and is transferred to the cytoplasm of the cell (Sandy & Butler, 2009 ▸; Chu *et al.*, 2010 ▸; Miethke, 2013 ▸; Schalk & Guillon, 2013 ▸; Delepelaire, 2019 ▸). This is followed by the release of iron either by degradation of the siderophore or by reduction of the iron(III) to iron(II) catalyzed by free extracellular or membrane-bound ferric chelate reductases (Hider & Kong, 2010 ▸).

**Figure 1:**
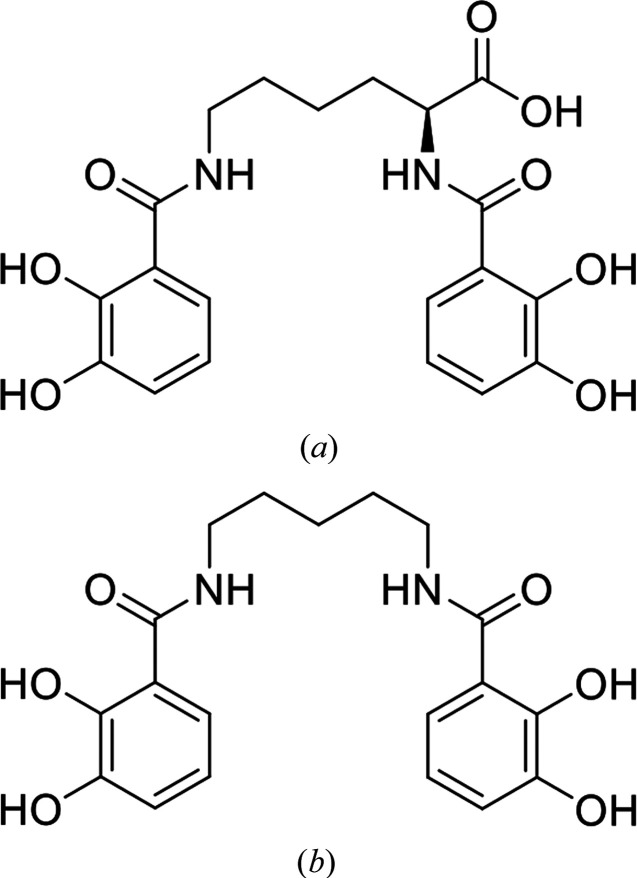
The chemical structures of (*a*) azotochelin and (*b*) 5-LICAM.

The periplasmic binding protein (PBP) CeuE is an important component of the iron(III)-uptake system in the Gram-negative bacterium *Campylobacter jejuni*, which does not itself synthesize siderophores but instead acquires iron by exploiting siderophores synthesized and secreted by other bacteria, such as *Escherichia coli*. *C. jejuni* can utilize a diverse range of catecholate siderophores for the uptake of iron(III), including tetradentate siderophores such as azotochelin and the enterobactin-derived bis(2,3-dihydroxybenzoyl-l-serine) (bisDHBS; Naikare *et al.*, 2013 ▸; Zeng *et al.*, 2013 ▸; Raines *et al.*, 2013 ▸; Zhang *et al.*, 2020 ▸). *Cj*CeuE binds a range of synthetic tetradentate enterobactin analogue ligands with high affinities. Crystal structures of *Cj*CeuE have been determined for iron(III) complexes with 4-LICAM (Raines *et al.*, 2013 ▸), 5-LICAM, 6-LICAM and 8-LICAM (Wilde *et al.*, 2017 ▸) and iron(III)-bisDHBS, which were synthesized to mimic the *N*,*N*′-bis(2,3-dihydroxybenzoyl)-*O*-seryl serine component of enterobactin (Raines *et al.*, 2016 ▸). The structures of azotochelin and 5-LICAM are shown in Fig. 1 ▸. We have previously used *Cj*CeuE as a protein scaffold in the design and production of an artificial metalloenzyme (ArM), in which iron(III)-azotochelin was used as an anchor to connect a synthetic iridium-based transfer hydrogenation catalyst to *Cj*CeuE, thereby creating an artificial transfer hydrogenase (Raines *et al.*, 2018 ▸). His227 and Tyr288 in *Cj*CeuE participate in the coordination of the iron(III) centre and are key amino acids for the binding of the iron complex of the tetradentate catecholate siderophore. Site-directed mutagenesis was used to show the relative contribution of His227 and Tyr288 to binding (Raines *et al.*, 2013 ▸, 2016 ▸, 2018 ▸; Wilde *et al.*, 2017 ▸).

Our current aim was to find a more thermostable siderophore-binding homologue containing the conserved histidine and tyrosine residues to enable ArM-catalyzed reactions to be performed at higher temperatures. In addition, we were looking for a homologue that retains its stability and siderophore-binding ability in the presence of organic solvents, in particular dimethylformamide (DMF), to facilitate the solubilization of hydrophobic siderophore–catalyst conjugates during ArM assembly and to extend the organic substrate scope of the catalytic reaction.

Two homologues of *Cj*CeuE were identified in the thermophilic Gram-positive bacteria *Geobacillus stearothermo­philus* and *Parageobacillus thermoglucosidasius*. These homologues are thought to be lipoprotein siderophore-binding proteins, but there is no experimental evidence to date that such proteins bind siderophores or synthetic siderophore analogues. Thermostable proteins tend to have the advantage of increased stability in a range of biotechnological applications. Here, we present the cloning, expression, purification and characterization of these proteins through biophysical and biochemical analyses and the determination of their crystal structures. The interactions between the *G. stearothermo­philus* and *P. thermoglucosidasius* proteins and the sidero­phore azotochelin and the synthetic azotochelin analogue iron(III)-5-LICAM are compared with the ligand-binding ability of *Cj*CeuE.

## 2. Materials and methods

### 2.1. Sequence-database search for thermophilic homologues of CeuE

A sequence-database search carried out using *BLAST* (Boratyn *et al.*, 2013 ▸) identified two homologues of *Cj*CeuE in the thermophilic Gram-positive bacteria *G. stearothermo­philus* and *P. thermoglucosidasius* (the proteins are referred to as Gst and Pth, respectively, in the following).

### 2.2. Cloning, expression and purification

The first crystal structures of *Cj*CeuE were of the apo protein and its complex with the siderophore analogue MECAM (Müller *et al.*, 2006 ▸). Initial crystallization trials used the full-length protein and were unsuccessful. It was decided to remove the N-terminal region including the signal peptide by mild proteolysis, which led to a fragment starting at Leu24, which was then successfully crystallized. The fragment which had been removed corresponded to the signal peptide and the first 23 residues of the mature protein, which are presumed to form an extended linker between the N-terminal Cys1 membrane anchor and the compact folded domain (residues 24–310). Subsequent structural studies used a construct corresponding to this ordered region to avoid the need for proteolytic cleavage of the disordered region. The constructs for Gst and Pth were selected to correspond to the equivalent regions of these proteins: residues 19–300 of the mature protein for Gst and residues 16–297 for Pth.

The synthetic DNA genes for Gst and Pth with optimized codon distribution and GC content were purchased from ThermoFisher Scientific and used as templates. Amplification of the DNA constructs starts at sites 39 and 37 for the Gst and Pth DNA, respectively, so that the signal peptide sequences have been removed. Mature Gst (19–300) and Pth (16–297) were directionally cloned into the LIC-adapted pET-28a vector (YSBLic3C) using the In-Fusion HD Cloning Kit [Clontech Laboratories, produced by Takara Biotechnology (Dalian)]. This vector contains an N-terminal hexahistidine tag linked to the gene of interest by a human rhinovirus 3C protease cleavage site that allows removal of the tag. Three additional residues remain in the protein (glycine, proline and alanine) after cleavage by 3C protease.

The genes for Gst and Pth were PCR-amplified using the primers shown in Supplementary Fig. S1 (YSBLic3C specific ends have been added to the primers and are shown in bold). Both proteins were expressed in *E. coli* strain BL21(DE3). Cells were grown with shaking at 37°C in Luria–Bertani broth containing 30 µg ml^−1^ kanamycin to an OD_600_ of 0.6–0.8 and were induced with 1 m*M* isopropyl β-d-1-thiogalactopyranoside for 3.5–4 h.

The buffers used for the isolation and purification of both proteins were buffer *A* (50 m*M* Tris–HCl pH 7.5, 500 m*M* NaCl, 10 m*M* imidazole), buffer *B* (50 m*M* Tris–HCl pH 7.5, 500 m*M* NaCl, 500 m*M* imidazole) and buffer *C* (20 m*M* Tris–HCl pH 7.5, 150 m*M* NaCl). The cells were harvested by centrifugation and resuspended in buffer *A* in the presence of cOmplete Mini, EDTA-free protease-inhibitor cocktail tablets (Roche Diagnostics GmbH, Germany) and were lysed by sonication on ice. The soluble crude extract was collected by centrifugation at 19 900 rev min^−1^. A standard three-step purification procedure was used for both Gst and Pth. Initially, a 5 ml HisTrap chelating column (Amersham Pharmacia) charged with nickel and equilibrated with buffer *A* was used. The elution buffer was buffer *B*. Fractions containing proteins of interest were eluted. His-tag cleavage was performed by adding 3C protease in a 1:100 ratio and the digest was dialysed overnight against buffer *C*. The second purification step was a run on a second Ni–NTA agarose column using buffer *C* to load the samples and buffer *B* for elution. From this step very pure nontagged protein eluted in the flowthrough fractions and less pure material, including traces of tagged protein and excess 3C protease, eluted in the low-gradient fraction. The flowthrough fractions were combined, concentrated by centrifugal ultrafiltration (Amicon Ultra) and run on a Superdex S200 column in buffer *C* (third purification step). After gel filtration, the final samples were concentrated to 100 mg ml^−1^ for Gst and 150 mg ml^−1^ for Pth. Concentrations were measured by the Bradford method (Coomassie Protein Assay Reagent, Thermo Scientific, USA). This resulted in pure proteins with very good yields (greater than 100 mg for both). The molecular mass was confirmed by electrospray mass spectrometry. These samples were used for crystallization and other characterization.

### 2.3. Biophysical characterization of the Gst and Pth proteins

#### 2.3.1. Electrospray mass spectrometry and circular dichroism (CD)

Electrospray mass spectrometry (ESI-MS) was used to confirm the molecular masses of the Gst and Pth proteins (Supplementary Fig. S2) and showed their experimental molecular weights to be in close agreement with the expected values. The correct folding of the proteins was confirmed using CD spectroscopy (Supplementary Fig. S3).

### 2.4. Thermostability screening

Thermostability assays were carried out using a Prometheus NT.48 differential scanning fluorimeter to measure the thermal unfolding of *Cj*CeuE, Gst and Pth, both unliganded and complexed with siderophore analogues. As reported previously (Wilde *et al.*, 2017 ▸), *Cj*CeuE is able to bind a range of synthetic ligands including tetradentate siderophores such as azotochelin and synthetic azotochelin analogue ligands, in particular iron-coordinated 5-LICAM^4−^. The interaction between the *G. stearothermophilus* and *P. thermoglucosidasius* proteins and the iron(III)-bound forms of the siderophore azotochelin and the synthetic azotochelin analogue 5-LICAM was compared with the substrate-binding ability of *Cj*CeuE.

To prepare the protein complexes, iron(III)-azotochelin or iron(III)-5-LICAM in DMF were added in a twofold excess to *Cj*CeuE, Gst or Pth and left to equilibrate for 30 min before washing out the excess of unbound ligand/DMF using a Falcon concentrating filter during centrifugation (10 000 molecular-weight cutoff). All proteins/complexes were diluted to ∼2.0–2.5 mg ml^−1^. A denaturation–unfolding cycle was used starting at 20°C and ending at 95°C with 1°C steps. An excitation power of 50% was used to obtain sufficient recordings for each protein. The fluorescence ratio (*F*_350_/*F*_330_) was recorded and the first derivative was plotted. The thermal unfolding transition midpoint (*T*_m_) was determined using the *PR.ThermControl* software.

### 2.5. Structure prediction with *AlphaFold*2

3D structures were predicted for the intact mature sequences of the *Cj*CeuE, Gst and Pth proteins employing the *ColabFold* server using the basic default parameters: https://colab.research.google.com/github/sokrypton/ColabFold/blob/main/AlphaFold2.ipynb#scrollTo=kOblAo-xetgx.

The pLDDT values used to estimate the confidence level of the prediction in *AlphaFold*2 (AF2) were converted to ‘*B* values’ within *CCP*4 to aid their use in molecular replacement and for display in *CCP*4*mg* (Baek *et al.*, 2021 ▸; Oeffner *et al.*, 2022 ▸). The generated structures were subsequently compared with the experimental X-ray structures and, in the case of Pth, used in the molecular-replacement structure solution.

### 2.6. Crystallization

#### 2.6.1. Apo Gst and apo Pth

Automated crystallization screening was performed in 96-well plates using a Mosquito nanolitre pipetting robot (SPT Labtech) by sitting-drop vapour diffusion with the PACT, Ammonium Sulfate, JCSG+ and Index screens. Each crystallization drop consisted of 150 nl protein solution and 150 nl reservoir solution. At protein concentrations of 20 and 40 mg ml^−1^ no crystals grew in the commercial screens. The protein concentrations were increased to 50 and 100 mg ml^−1^. Initially, crystals that were not ideal only appeared after three months in the JCSG+ screen (condition E6) for Pth and in the Index (conditions D3 and G6) and JCSG+ (condition G1) screens for Gst. These were thin clustered plates which were crushed and used as seeds for another round of screening using commercial screens and optimization using an Oryx8 Protein Crystallization Robot (Douglas Instruments), which mixes 0.05 µl seed solution, 0.15 µl protein solution (at the same protein concentration as used in the initial screen) and 0.1 µl well solution. The conditions used for the apo Pth and Gst crystals used for X-ray data collection are shown in Supplementary Table S1.

#### 2.6.2. Preparation of Pth and Gst complexes for crystallization

Iron(III)-azotochelin and iron(III)-5-LICAM were synthesized according to a published procedure (Raines *et al.*, 2013 ▸) to obtain purple solids, which were dissolved in dimeth­ylformamide to give a 50 m*M* stock solution of each ligand. For co-crystallization, a solution of Gst or Pth was diluted to 20 mg ml^−1^ in a buffer consisting of 20 m*M* Tris–HCl, 150 m*M* NaCl pH 7.5 and then mixed with the ligand stock solution in a 1:10 molar ratio. After adding the appropriate volume of ligand solution, the mixture was kept for 10–20 min at room temperature. The resulting protein–ligand mixture was then centrifuged at 13 000 rev min^−1^ for 2–3 min to remove any precipitant. To wash out the excess ligand and DMF, additional buffer was added and the diluted solution was re-concentrated using Amicon centrifugal filter units. Several washes in concentrating units (dilution–concentration) were performed. The first flowthrough solutions were coloured, while the final flowthrough solutions were not. The protein complex solution for co-crystallization was finally concentrated to 40–50 mg ml^−1^ (measured by the Bradford method).

Gst was also co-crystallized in complex with a synthetic azotochelin-iridium catalyst similar to that reported in a previous publication (Raines *et al.*, 2018 ▸). The protein and ligand were mixed in a 1:1 ratio, diluted with additional 50 m*M* Tris–HCl, 0.15 *M* NaCl pH 7.5 buffer, washed and concentrated for crystallization. The catalytic iridium-containing moiety was hydrolyzed during the time required for crystal growth and only azotochelin was observed to be bound to the protein.

#### 2.6.3. Pth and Gst complex crystallization

As for the apoproteins, automated crystallization screening for the protein–ligand complexes was performed in 96-well plates with an Oryx8 Protein Crystallization Robot by sitting-drop vapour diffusion using the commercial PACT (Molecular Dimensions) and JCSG+ screens. Seeds (50 nl) prepared from apoprotein crystals were added to each drop consisting of 150 nl complex solution and 100 nl reservoir solution. After the seeds had been introduced, crystals grew in 5–10 days and were coloured. Crystals of the Gst–iron(III)-5-LICAM, Pth–iron(III)-azotochelin and Pth–iron(III)-5-LICAM complexes were obtained under a number of conditions in the commercial screens and optimizations. The diffraction quality of crystals grown in different conditions was tested in-house. The crystallization conditions of the best diffracting crystals used for structure solution are shown in Supplementary Table S1. All apo and complex crystals were obtained using microseed matrix screening (MMS; for a review, see D’Arcy *et al.*, 2014 ▸). In all cases the seeds were prepared from apo crystals.

#### 2.6.4. X-ray structure solution

Data were collected on beamlines at Diamond Light Source (DLS). Computations were carried out using programs from the *CCP*4 suite (Agirre *et al.*, 2023 ▸). Where appropriate, the structures were solved by molecular replacement with *MOLREP* (Vagin & Teplyakov, 2010 ▸). They were refined with alternating cycles of *REFMAC*5 (Murshudov *et al.*, 2011 ▸) and *Coot* (Emsley *et al.*, 2010 ▸). The data-collection and refinement statistics are shown in Table 1 ▸.

The crystal structure of apo Gst was solved by molecular replacement using the structure of *Cj*CeuE as a search model and rebuilt with *Buccaneer*. The structures of the complexes of Gst with iron(III)-azotochelin and iron(III)-5-LICAM were solved using the apoprotein as a search model. All three structures are of reasonable quality, as can be seen from the statistics in Table 1 ▸.

For Pth, the structure of the azotochelin complex was first solved using the *AlphaFold*2-predicted model for the residues in the construct as the molecular-replacement search model. The resulting protein model only required minimal rebuilding of a small number of side chains; the main chain required essentially no rebuilding, with the exception of a couple of peptide flips. The 5-LICAM complex was isomorphous to the azotochelin complex and was built starting from the model of the azotochelin complex. The apo Pth structure was isomorphous to the two ligand complexes and was built starting from the protein component of the azotochelin complex. The structures were of moderate quality, reflecting the difficulty in obtaining diffraction-quality crystals, and as can be seen from Table 1 ▸ the mean *B* values are high for the Pth structures.

### 2.7. Binding-affinity determination by intrinsic fluorescence quenching

Fluorescence spectra were recorded on a Hitachi F-4500 fluorescence spectrophotometer with an excitation wavelength of 280 nm, an emission range of 295–410 nm, an excitation slit width of 10 nm, an emission slit width of 20 nm, a scanning speed of 60 nm min^−1^, automatic response, corrected spectra and a detector voltage of 950 V. Stock solutions of *Cj*CeuE, Gst, Pth, iron(III)-5-LICAM and iron(III)-azotochelin were prepared in buffer (40 m*M* Tris–HCl pH 7.5, 150 m*M* NaCl). Stock concentrations were optimized for each protein/ligand pair to reach a homogeneous data distribution. For each replicate, 2 ml protein solution was placed in a 1 cm quartz cuvette and titrated stepwise (1 µl per step) with ligand solution using a DOSTAL DOSY liquid dispenser (loaded with 20 µl ligand solution) with continuous stirring at room temperature. After each addition, the solution was allowed to equilibrate for 1 min before scanning. Each system was analysed in triplicate or duplicate as indicated. Fluorescence spectra were buffer-subtracted and integrated between 310 and 380 nm, with the normalized peak area plotted as a function of ligand concentration using *Origin* 2021b. *K*_d_ values were obtained by fitting the experimental data to equation (1) (adapted from Jiang *et al.*, 2019 ▸) using the *Origin* user-defined nonlinear curve-fitting analysis,

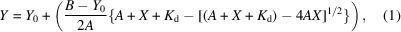

where *Y* is the normalized fluorescence emission, *Y*_0_ is the initial normalized fluorescence emission (before any ligand addition), *B* is the minimum normalized fluorescence emission (fully quenched state), *A* is the protein concentration and *X* is the ligand concentration.

The protein concentrations were determined using the Beer–Lambert law based on the absorbance at 280 nm and the following corrected theoretical molar extinction coefficients: ɛ_*Cj*CeuE_ = 18 590 cm^−1^ *M* ^−1^, ɛ_Pth_ = 29 196 cm^−1^ *M* ^−1^ and ɛ_Gst_ = 34 239 cm^−1^ *M* ^−1^ (determined as described in the supporting information, Supplementary Fig. S8 and Supplementary Table S2).

To investigate the organic solvent tolerance of the three proteins, analogous fluorescence-quenching experiments were carried out in buffer mixtures that contained 10% and 20% dimethylformamide (DMF). Reference curves with 0% DMF were collected in parallel and the respective *K*_d_ values obtained were used for normalization.

## 3. Results and discussion

### 3.1. Identification of two thermophilic homologues of CeuE

**Figure 2:**
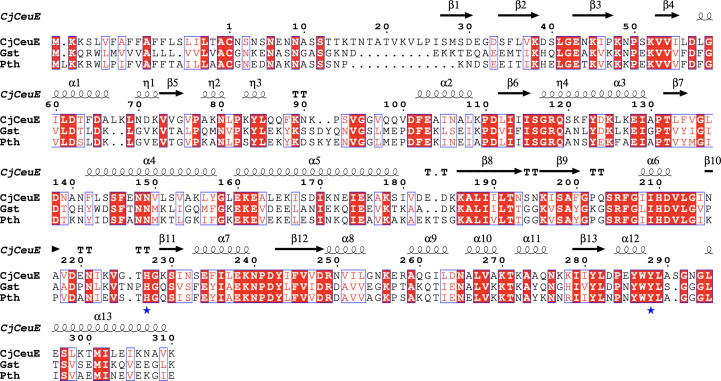
Amino-acid sequences of the proteins. (*a*) Alignment of full-length *Cj*CeuE, Gst and Pth using the *T-Coffee* server at the EBI. The secondary structure of *Cj*CeuE is shown above the sequences. The conserved histidine and tyrosine residues that are important in the binding of tetradentate siderophores are indicated by blue asterisks.

Gst and Pth have almost 50% sequence identity to *Cj*CeuE (and 68% to one another) and contain the His227 and Tyr288 residues that are involved in tetradentate siderophore binding in *Cj*CeuE (Fig. 2 ▸). All three proteins have a signal peptide at the N-terminus, which is cleaved between the Ala and Cys residues (numbers 0 and −1 in *Cj*CeuE) after secretion from the cell (Supplementary Fig. S4). Our numbering of all three starts from the N-terminus of the mature protein. The terminal cysteine in the mature protein is important as it allows attachment of the protein to the membrane via the addition of palmitic acid to the cysteine (Richardson & Park, 1995 ▸). Constructs of the mature proteins were successfully cloned and expressed as described in Section 2. The sequences of the constructs are shown in Supplementary Fig. S4.

### 3.2. Thermostability

Thermostability assays were carried out as described in Section 2. The results are shown in Supplementary Fig. S5. Apo Pth and Gst have a higher thermal unfolding transition midpoint (*T*_m_) and are substantially more thermostable than *Cj*CeuE. The *T*_m_ of Pth is 82.5°C and that of Gst is 80.9°C, while the *T*_m_ of *Cj*CeuE is 60.4°C (Table 2 ▸). The refolding phase showed no points of inflection: once the proteins have been denatured they do not refold into their native forms when the temperature is decreased back to 20°C. As evident in Table 2 ▸, the *T*_m_ values increased on ligand binding for all three proteins. In some cases we observe two or even three shifts of *T*_m_ caused by ligand binding which might reflect nonspecific binding of the ligand or show the appearance of species with a stoichiometry other than 1:1.

### 3.3. Structures of the thermophilic proteins

#### 3.3.1. Crystal structures

**Figure 3:**
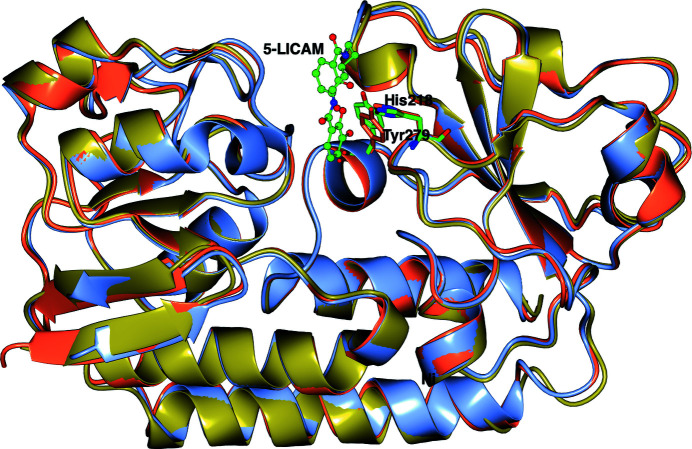
Superposition of the three Gst structures in ribbon format: apo protein (ice blue), azotochelin complex (gold) and iron(III)-5-LICAM complex (coral). Iron(III)-5-LICAM is shown in ball-and-stick representation coloured by atom type, with the iron-chelating residues as cylinders (azotochelin is not shown to simplify the view but superimposes closely on 5-LICAM). Tyr279 and His218 are shown in green for the apo protein; His218 is very close to its position in the ligand complexes.

All numbers refer to the mature sequences without the signal peptide. As expected, the overall fold of both Gst and Pth is very similar to that of *Cj*CeuE and is typical of such periplasmic binding proteins. The r.m.s.d. from apo *Cj*CeuE is 1.26 and 1.48 Å for apo Gst and Pth over 266 and 272 structurally equivalent C^α^ atoms, respectively, while that between Gst and Pth is 0.82 Å over 273 C^α^ atoms. The proteins have a two-domain structure, with the domains linked by a long α-helix at the base of the fold. The siderophore binding sites sit between the two domains. The crystal structure of apo Gst was solved by molecular replacement using the structure of *Cj*CeuE as a search model. There is a single molecule in the asymmetric unit with a continuous chain from Met26 through to the C-terminal Lys300. The structures of the complexes of Gst with azotochelin and 5-LICAM were solved using the apo protein as a search model. All three structures are of reasonable quality as can be seen from the statistics, reflecting the difficulty in obtaining diffraction-quality crystals. In the complex with azotochelin there was a single protein complex in the asymmetric unit, with density from Glu25 to Lys300, while the crystals of the complex with 5-LICAM contained two independent complexes both with density for residues Glu24–Lys300. The ligand structures superimpose closely on the apo model, with r.m.s.d.s over 273 equivalent C^α^ atoms of 0.61 Å for the azotochelin complex and 0.48 Å for the 5-LICAM complex (Fig. 3 ▸). Several of the loop regions on the surface of the protein have high *B* values and show high flexibility in all three structures, with some variation between the three. However, the regions around the siderophore site are well ordered. The conserved iron-chelating residues in the three structures are His227 and Tyr288 in *Cj*CeuE, His218 and Tyr279 in Gst and His215 and Tyr276 in Pth.

**Figure 4:**
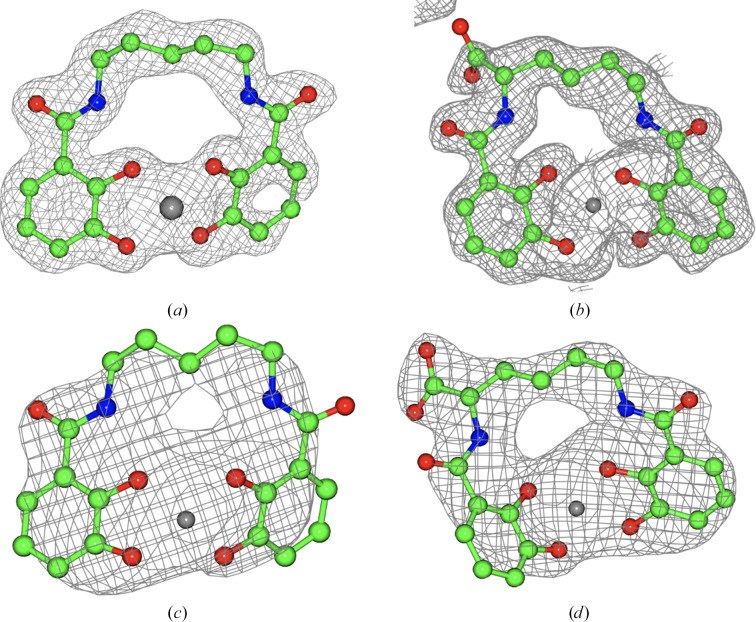
Difference electron density for the ligands. The models shown in ball-and-stick representation are the final refined structures. The difference density, clipped around the ligands, is for the maximum-likelihood maps before introduction of the ligand or iron ion into the models. The maps are contoured at levels of between 0.2 and 0.3 e Å^−3^, reflecting the resolution of each structure. (*a*) Gst–iron(III)-5-LICAM, (*b*) Gst–iron(III)-azotochelin, (*c*) Pth–iron(III)-5-LICAM, (*d*) Pth–iron(III)-azotochelin. There is a significant anomalous density peak at each of the iron positions (not shown).

**Figure 5:**
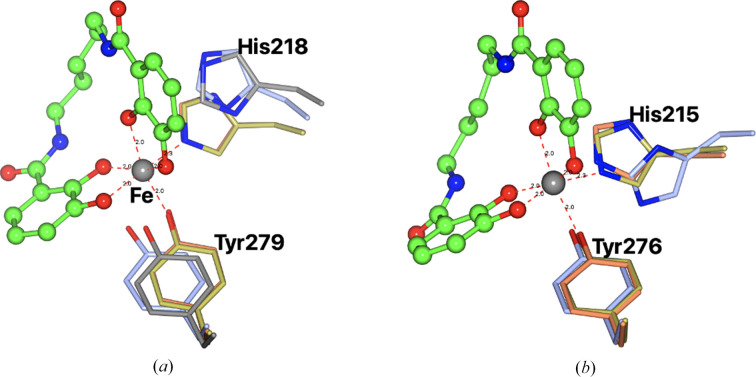
The ligand-binding histidine and tyrosine residues in the Gst and Pth structures. The experimental apo structure (ice blue C atoms), the 5-LICAM complex (gold), the azotochelin complex (coral) and the model predicted by AF2 (grey) are shown. (*a*) Gst, (*b*) Pth. The AF2 model is only shown for Gst. The positions of the histidine and tyrosine residues superimpose so closely that they are hard to distinguish in (*a*).

As expected, the Fe atom of the ligand in both Gst complexes is bound by the four catecholate O atoms and the conserved His218 and Tyr279. The electron density for the ligands is shown in Figs. 4 ▸(*a*) and 4 ▸(*b*). The position of the His218 loop is shown in Fig. 5 ▸. The main chain carrying these residues is in essentially the same conformation in the experimental and predicted models. This is in contrast to the apo *Cj*CeuE structure, in which the equivalent His227 loop moves away from its iron-binding position in the complexes, probably allowing easy access for the siderophore-like ligands. The side chains of the two residues in the two complexes are seen to superimpose very closely, and differ slightly from their position in the experimental and AF2-modelled apo structure. Thus, the AF2 model has accurately predicted the fold in the experimental apo structure around the ligand-binding site. It should of course be noted that these structures are from crystals cryogenically frozen at 100 K and that in natural Gst cells at their optimum growth temperature of around 65°C the His218 loop might well open up, as seen in *Cj*CeuE.

All three Pth structures have considerably higher *B* values than those for Gst, with the azotochelin complex being the best ordered of the three. The three structures are closely similar to one another, with r.m.s.d.s of 0.43 and 0.48 Å compared with the apo form for the 5-LICAM and azotochelin complexes, respectively. For Gst, the structure of the azotochelin complex was obtained from co-crystallization with an iridium catalyst complex similar to that reported for *Cj*CeuE. However, it appears that the catalyst moiety cleaved during the crystal-growth period and density was only visible for the residual azotochelin. This structure was of considerably better quality than that from a co-crystal of Gst with azotochelin alone and hence was chosen for detailed analysis. The structure was solved using the AF2-predicted model (see below) of the residues in the construct as the molecular-replacement search model. The resulting protein model required only minimal rebuilding of a small number of side chains. The main chain required essentially no rebuilding, with the exception of a couple of peptide flips. There was clear density for the ligand (Fig. 4 ▸ *d*). The 5-LICAM complex was isomorphous to the azotochelin complex and was built starting from the azotochelin complex as a model; it again showed clear density for the ligand (Fig. 4 ▸ *c*). The apo Pth structure was essentially isomorphous to the two complexes and was again built starting from the protein component of the azotochelin complex. In contrast to the Gst–azotochelin complex, the Pth–azotochelin complex was obtained by co-crystallization with iron(III)-azotochelin.

In all three isomorphous structures there were difference density features associated with clear peaks in the anomalous difference maps which were modelled as nickel ions. While the nickel ions are not functionally significant, a brief description of them is given here. The numbers refer to the positions of the Ni atoms in the chains in the deposited PDB files in the order apo, 5-LICAM complex, azotochelin complex: Ni1 (B1, A309, A305), Ni2 (B2, A307, A310), Ni3 (B3, A308, absent), Ni4 (B4, A306, A311), Ni5 (B5, absent, A312). There is also a sulfate ion (C1, A310, A314). The Ni1 and Ni4 sites in each structure lie on a crystallographic twofold axis and are linked through the sulfate ion. The Ni–SO_4_–Ni moiety sits between two adjacent protein molecules in the crystal lattice: one of these nickel ions is coordinated by Glu94 and its symmetry-related mate and the second is coordinated by Asp88 and its symmetry-related mate. Another nickel ion is coordinated by Glu151 and Glu154 and their symmetry equivalents. Ni2 is also on a twofold axis but does not have an associated sulfate ion. Ni3 and Ni5 are associated with the same protein side chains in the three structures but are remote from the siderophore binding site and do not interfere with ligand binding. These nickel cations and the associated sulfate anion are assumed to have been carried over from the nickel purification column. As will be seen below, they do not appear to affect ligand binding in solution significantly.

#### 3.3.2. Structure and thermostability

The mature proteins are roughly 280 amino acids in length and those from the thermophiles, while about 70% identical to one another, are only 50% identical to *Cj*CeuE. Hence, it is not straightforward to identify the features of the two thermophilic proteins that are responsible for their thermostability. The secondary-structural elements are essentially identical in *Cj*CeuE and the thermophilic proteins; there are no major difference in loop sizes, and the number of charged residues and salt-bridge interactions are roughly similar in all three proteins. Calculation of hydrophobic clusters using the *ProteinTools* server (Ferruz *et al.*, 2021 ▸) did not indicate that there were additional clusters in the thermophilic proteins. Computation with the Expasy *ProtParam* tool (Wilkins *et al.*, 1999 ▸) based on the sequences of the ordered part of the structures rather than the 3D structures resulted in a grand average of hydropathicities (GRAVY) of −0.145 for *Cj*CeuE, −0.393 for Gst and −0.319 for Pth, suggesting a small increase in hydrophobicity overall for the thermophilic proteins.

Examination of the fit between the thermophilic proteins and *Cj*CeuE showed a few regions which deviated more significantly. These are roughly residues (*Cj*CeuE numbering) 96–99, 220–240 (the His227 ligand-binding loop), 256–266 and 289–293. The most significant changes in hydrogen bonding are in the ligand-binding loop. In the thermophilic proteins this is tightly bound to the rest of the structure (with several strong hydrogen bonds) and is close to its position after ligand binding. In *Cj*CeuE this is not the case. The most significant sequence difference is at residue 200 of *Cj*CeuE, which is a phenylalanine but is equivalent to a tyrosine in the thermophilic proteins. The hydroxy group of tyrosine in these structure helps to anchor the loop.

A more extensive analysis would be required to analyse such differences in depth, requiring bioinformatics to identify pairs of residues that are present in the thermophilic proteins but are absent in the mesophilic proteins, followed by site-directed mutagenesis of these amino acids and measurement of the effect of the mutations on the thermostability. Such a programme of work, however, was not within the scope of the present study, which aimed at the identification of thermophilic CeuEs for further work in artificial metalloenzyme development.

#### 3.3.3. *AlphaFold*2 predictions

**Figure 6:**
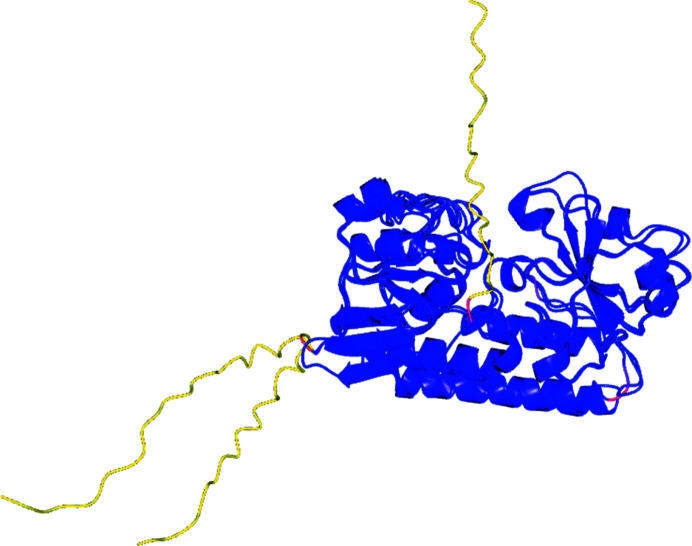
3D structures of the mature proteins as predicted using the *ColabFold* *AlphaFold*2 server. The structures are coloured from blue (confident prediction) through red (medium confidence) to yellow (not confident).

The structures of the mature proteins predicted using AF2 are shown in Fig. 6 ▸. The three structures superimpose very closely for the well predicted blue regions, with an r.m.s.d. between equivalent C^α^ atoms of between 0.8 and 0.9 Å, as might be expected since the X-ray structure of *Cj*CeuE is already present in the PDB and there are an extensive number of sequences of homologues in the public database used by AF2. The extended tails (yellow) leading to the N-terminal cysteine involved in linking the protein to the cell wall are predicted as having very low positional confidence and can be assumed to be flexible. The predictions confirm that a sensible choice had been made for the constructs expressed for crystallization, with the blue confident predictions starting around Val23 in *Cj*CeuE, Glu25 in Gst and Glu20 in Pth.

Superposition of the experimental structures on the AF2 models confirmed how good the latter were. The predicted and experimental apo Gst structures had an r.m.s.d. between all 273 C^α^ atoms of 0.62 Å. The r.m.s.d. for the Pth structures was 0.65 Å. The value for *Cj*CeuE is not meaningful as the structure is already present in the PDB. What is of note is that AF2 predicts the His227 loop to be in the open conformation as in the experimental structure of apo *Cj*CeuE, with residue 227 swung out away from the iron siderophore-binding position. The histidine loop in *Cj*CeuE is indicated to be somewhat flexible by AF2. In Gst and Pth, the position of the main chain of the histidine loop is closely similar in the apo, complex and predicted structures, with the histidine side chain having moved a couple of ångströms away from its iron-binding position in the apo and AF2 structures.

### 3.4. Fluorometric determination of ligand-binding affinity

The binding curves obtained by intrinsic fluorescence-quenching experiments are shown in Supplementary Fig. S6. Data fitting provided the binding constants given in Table 3 ▸. With a value of ∼18 n*M*, the *K*_d_ obtained for the binding of iron(III)-5-LICAM to *Cj*CeuE is slightly higher than the previously estimated value of <10 n*M* (Wilde *et al.*, 2017 ▸). We believe that the results reported here are more accurate for three reasons: (i) the theoretical molar extinction coefficient was corrected for the denatured protein, (ii) the fitting method used took into account that the fully bound state still gives rise to a baseline level of fluorescence (not tryptophan related) and (iii) the titration procedure was carried out using an automated titrator (DOSY).

It was found that the two thermophilic homologues Gst and Pth bind iron(III)-5-LICAM about tenfold more strongly than *Cj*CeuE, while the affinity for iron(III)-azotochelin is similar for all three proteins.

**Figure 7:**
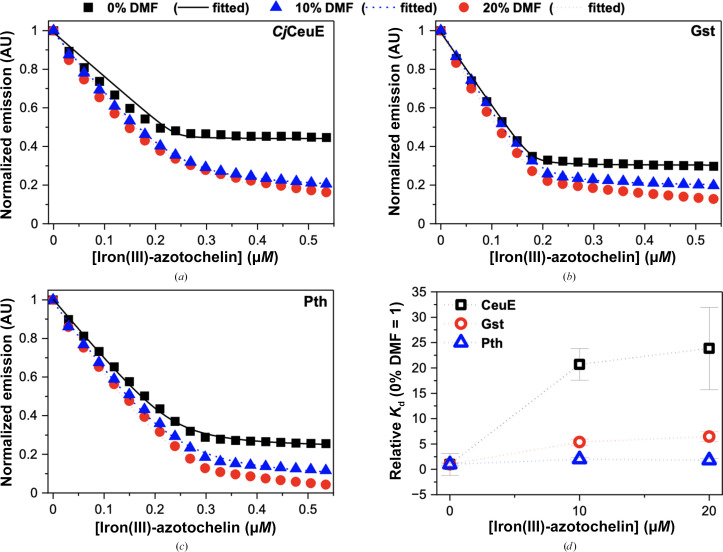
Effect of DMF on the affinity of the three proteins for iron(III)-azotochelin. The binding curves for *Cj*CeuE (*a*), Gst (*b*) and Pth (*c*) were obtained by intrinsic fluorescence quenching in the absence of (black squares) and the presence of 10% (blue triangles) and 20% (red circles) DMF. The *K*_d_ values were estimated from the fitted curves using equation (1) and expressed relative to their respective 0% DMF control (*d*). The buffer was 40 m*M* Tris–HCl pH 7.5, 150 m*M* NaCl. Curves were collected in duplicate and averaged. Data with respective mean absolute deviations are available in Supplementary Fig. S7.

To evaluate whether the improved thermostability and siderophore-binding affinity of Gst and Pth concurs with an increase in solvent tolerance, fluorescence-quenching assays were carried out in the presence of increasing amounts of an organic solvent. Tolerance to organic solvents could play a major role in the application of these homologues, for example in the development of artificial metalloenzymes. We selected DMF due to its biological compatibility and water miscibility and because the siderophores studied here are highly soluble in this solvent. As expected, the siderophore-binding affinity of all three proteins was found to decrease with an increase in the percentage of DMF in the buffer mixture. This could be due to conformational changes, partial protein unfolding triggered by DMF or improved solvation of the relatively hydrophobic siderophore ligands in the more hydrophobic solvent shifting the binding equilibrium. Whilst the addition of DMF leads to a very notable change in the shape of the binding curve obtained with *Cj*CeuE (Figs. 7 ▸ *a*–7 ▸ *c*), the changes observed with Gst and Pth are less pronounced. The corresponding *K*_d_ values were estimated (equation 1; fitted curves are shown in Figs. 7 ▸ *a*–7 ▸c), normalized using the respective *K*_d_ values obtained in the absence of DMF and plotted in Fig. 7 ▸(*d*). It is evident that *Cj*CeuE is drastically affected, with the binding affinity decreasing by about 20-fold at 10% DMF and 25-fold at 20% DMF. Pth, on the other hand, remains relatively stable, with a less than twofold decrease in the binding affinity in the presence of either 10% or 20% DMF. The solvent tolerance of Gst is slightly lower than that of Pth, with an approximately fivefold increase in *K*_d_ observed at 20% DMF.

The binding affinity of *Cj*CeuE for 5-LICAM is a little low compared with the other cases (Table 3 ▸). We originally rationalized the difference in the affinity of *Cj*CeuE for 5-LICAM and azotochelin by the presence of the extra carboxyl group on azotochelin (missing in 5-LICAM), which lies quite close (about 3.7 Å) to Arg249. However, this is also true for the thermophilic proteins, where there is an even shorter ionic interaction between the azotochelin carboxyl and the equivalent arginine, but there is no difference in binding energy between azotochelin and 5-LICAM. What should be noted is that the difference in binding constant is only a factor of four, which corresponds to a difference of about 3 kJ mol^−1^ in Δ*G*. This is a very small difference (around a third of a typical hydrogen bond). A potential entropic effect, however, is worth noting. The histidine iron-binding loop (His227 loop in *Cj*CeuE) is in two alternate conformations in apo *Cj*CeuE, suggesting that it is partly open in the apo structure, allowing ready access to the siderophore. In contrast, the histidine loop is already in the closed, iron-chelating position in the apo structures of the thermophilic proteins. This is related to our observations on the thermostability above, where the fact that the histidine loop is more anchored/rigid in the thermophiles was also highlighted. Consequently, we have less of a ‘loss of entropy’ upon ligand binding in the thermophilic proteins and hence the *K*_d_ values for azotochelin and 5-LICAM are similar. In contrast, in *Cj*CeuE the hydrogen bond to the carboxylate group of azotochelin is more significant as an interaction that stabilizes (or arrests) the flexible histidine loop. To further complicate matters, the structures are all derived from proteins obtained at room temperature and then flash-cooled to 100 K for data collection. It might be expected that the thermophilic apo proteins also have an open conformation available to the histidine loop at the higher growth temperature of the organisms. It is perhaps not surprising that it is hard to relate the small difference in *K*_d_ for *Cj*CeuE directly to the structures.

## 4. Summary and conclusion

Genes coding for periplasmic binding proteins homologous to the well characterized CeuE from *Campylobacter jejuni* were identified from sequence databases. Synthetic genes coding for expected ordered regions of the proteins were purchased, cloned in *E. coli* strains, overexpressed and purified. The proteins from the thermophiles were shown to be correctly folded and to have melting temperatures about 20°C higher than that of *Cj*CeuE. Crystal structures were solved of the resulting proteins and of their complexes with iron(III)-azotochelin and iron(III)-5-LICAM. Crystallization proved to be more challenging than anticipated, and the resulting crystals were of only moderate quality. Nevertheless, there was clear density for the ligands and the protein chains were generally well ordered, with the exception of a few surface loops with poor density. In the Pth structures in particular, bound nickel and Ni–SO_4_–Ni moieties were modelled in all crystal forms at essentially the same sites as ligated by carboxylate side chains. The Fe atoms were coordinated by four O atoms from the two catecholate units of each ligand and the N and O donor atoms of conserved histidine and tyrosine residues, respectively, from the proteins. The binding constants were measured by fluorescence-quenching titrations and confirmed that the ligands were tightly bound with *K*_d_ values in the low-nanomolar range, as for *Cj*CeuE. In the presence of 10% and 20% DMF the respective binding affinities decreased notably for *Cj*CeuE but only slightly for Pth and Gst, indicating an improved organic solvent tolerance of the thermophilic homologues. *AlphaFold*2 was used to predict the structures using default parameters and the overall conclusion is that it predicted high-quality structures for the thermophilic proteins, probably reflecting the fact that the structures of *Cj*CeuE and several homologues are already present in the PDB. The predicted structures were very similar to the experimental structures in both fold and the position of side chains. In addition, the *AlphaFold*2 models highlighted the point at which the N-terminal region of the mature protein was likely to be ordered and suitable for crystallization, confirming that the chosen constructs were appropriate. The two thermophilic homologues Gst and Pth provide excellent possibilities for further development of these periplasmic proteins as scaffolds for artificial metalloenzymes, as we have demonstrated previously for *Cj*CeuE (Raines *et al.*, 2018 ▸), due to their increased thermostability and enhanced solvent tolerance.

## Supplementary Material

PDB reference: CeuE homologue from *Geobacillus stearothermophilus*, apo, 8b7x

PDB reference: complex with 5-LICAM, 8baw

PDB reference: complex with azotochelin, 8bax

PDB reference: CeuE homologue from *Parageobacillus thermoglucosidasius*, apo, 8bnw

PDB reference: complex with 5-LICAM, 8bj9

PDB reference: complex with azotochelin, 8bf6

Supplementary information, Supplementary Figures and Supplementary Tables. DOI: 10.1107/S2059798323004473/rr5232sup1.pdf

## Figures and Tables

**Table 1:** X-ray data-collection and refinement statistics

	Gst, apo	Gst–iron(III)-azotochelin	Gst–iron(III)-5-LICAM	Pth, apo	Pth–iron(III)-azotochelin	Pth–iron(III)-5-LICAM
Beamline	I03, DLS	I03, DLS	I03, DLS	I03, DLS	I04, DLS	I03, DLS
Wavelength (Å)	0.9762	0.9763	0.9762	0.9763	0.9795	0.9762
Temperature (K)	100	100	100	100	100	100
Space group	*P*1	*P*2_1_2_1_2_1_	*P*2_1_	*C*222_1_	*C*222_1_	*C*222_1_
*a*, *b*, *c* (Å)	32.519, 35.725, 52.290	34.158, 66.652, 117.056	67.395, 34.432, 119.109	35.369, 118.433, 141.046	36.295, 116.711, 141.471	36.063, 117.392, 141.712
α, β, γ (°)	81.57, 83.24, 65.06	90, 90, 90	90, 92.88, 90	90, 90, 90	90, 90, 90	90, 90, 90
Resolution range (Å)	32.20–1.42 (1.44–1.42)	57.53–1.38 (1.40–1.38)	67.31–1.47 (1.50–1.47)	70.52–2.13 (2.19–2.13)	70.74–1.97 (2.02–1.97)	38.80–2.07 (2.13–2.07)
*R*_meas_	0.034 (0.598)	0.136 (4.568)	0.048 (3.325)	0.076 (3.548)	0.027 (2.911)	0.056 (3.592)
*R*_p.i.m._	0.021 (0.390)	0.037 (1.249)	0.010 (1.304)	0.021 (0.979)	0.021 (0.008)	0.013 (1.907)
Total reflections	76272 (2925)	739510 (36790)	606557 (29154)	216697 (17743)	280239 (20239)	437444 (18520)
Unique reflections	37722 (1729)	54541 (2598)	93663 (4020)	17078 (1377)	21889 (1526)	18904 (1420)
〈*I*/σ(*I*)〉	5.8 (0.8)	7.4 (1.0)	8.2 (0.5)	17.8 (0.5)	16.4 (0.9)	13.3 (0.5)
CC_1/2_	0.998 (0.862)	0.998 (0.473)	0.999 (0.432)	1.000 (0.377)	1.000 (0.408)	0.998 (0.238)
Completeness (%)	94.9 (88.8)	97.3 (95.4)	99.8 (99.2)	100.0 (100.0)	100.0 (99.5)	99.7 (98.7)
Multiplicity	2.1 (1.7)	13.6 (14.2)	6.5 (6.3)	12.7 (12.9)	12.8 (13.3)	13.3 (13.0)
Wilson *B* factor (Å^2^)	18.26	19.49	27.04	64.63	50.90	64.52
No. of reflections (working/test set)	37657/1940	54461/2753	93643/4698	17045/862	21853/1084	18940/938
Final *R*_cryst_	16.9	21.4	17.4	22.2	20.5	20.9
Final *R*_free_	24.0	26.2	22.8	26.6	25.5	26.8
R.m.s.d.s						
Bond lengths (Å)	0.0127	0.0086	0.0086	0.0083	0.0109	0.0100
Angles (°)	1.782	1.395	1.342	1.246	1.348	1.265
Cruickshank DPI	0.0937	0.0841	0.0842	0.2614	0.18785	0.2185
No. of non-H atoms						
Average *B* factors (Å^2^)	27.2	32.4	Chain *A*, 41.5; chain *B*, 48.4	78.6	43.28	87.3
*MolProbity* score	1.63	1.66	1.97	2.34	2.03	2.39
Ramachandran statistics						
Most favoured (%)	97.81	97.85	96.17	94.57	96.74	94.20
Outliers (%)	0.0	0.0	0.0	0.72	0.36	6.36
PDB code	8b7x	8bax	8baw	8bnw	8bf6	8bj9

**Table 2:** *T*_m_ values of *Cj*CeuE, Gst and Pth both unliganded and in complex with iron(III)-azotochelin and iron(III)-5-LICAM

	*T*_m_ (°C)		
	*Cj*CeuE	Gst	Pth
Apo protein	60.4	80.9	82.5
Complex with iron(III)-azotochelin	70.2	86.6	83.1, 88.6, 91.5
Complex with iron(III)-5-LICAM	67.6	83.6, 90.2	84.0, 90.7

**Table 3:** *K*_d_ values as measured by fluorescent titration for the binding of iron(III)-5-LICAM and iron(III)-azotochelin to the three proteins in 40 m*M* Tris–HCl pH 7.5, 150 m*M* NaCl

Ligand	*K*_d_ (n*M*)		
	*Cj*CeuE	Gst	Pth
Iron(III)-5-LICAM	17.9 ± 0.3	1.7 ± 0.3	3.3 ± 0.4
Iron(III)-azotochelin	4.9 ± 0.7	3.5 ± 0.7	5.3 ± 0.4
