# Supplementary material for: Thermostable homologues of the periplasmic siderophore-binding protein CeuE from Geobacillus stearothermophilus and Parageobacillus thermoglucosidasius
Source: Acta Crystallogr D Struct Biol. 2023 Jul 10;79(Pt 8):694–705. doi: 10.1107/S2059798323004473 (PMC10394670; doi:10.1107/S2059798323004473)
Supplement: Supplementary file 1 [file d-79-00694-sup1.pdf]

STRUCTURAL BIOLOGY

Volume 79 (2023)

Supporting information for article:

Thermostable homologues of the periplasmic siderophore-binding protein CeuE from *Geobacillus stearothermophilus* and *Parageobacillus thermoglucosidasius*

Elena V. Blagova, Alex Miller, Megan Bennett, Rosalind Booth, Eleanor J. Dodson, Anne-Kathrin Duhme-Klair and Keith S. Wilson

The genes for Gst and Pth were amplified PCR using the following primers.

FWD Gst 19 **TCCAGGGACCAGCA**ACCGAACAGGCAGAAGAGATG
REV Gst 300 **TGAGGAGAAGGCGCGTTA**TTTCAGGCCTTCTTCAACCTGT
FWD Pth 16 **TCCAGGGACCAGCA**AAAAATGATAGCGAAGAAATCAC
REV Pth 297 **TGAGGAGAAGGCGCGTTA**TTCGATGCCTTTTTCGACTTC

Figure S1 Primers used for PCR amplification, with YSBLic3C specific ends added to the primers shown in bold.

ESI-MS was used to determine the molecular masses of Pth and Gst (Bruker maXis HD qToF mass spectrometer). The protein samples were transferred to 2 mM TRIS buffer pH 7.5 using dialysis and concentrations of about 4 mg/ml were used for the measurements. The theoretical molecular masses for the Pth and Gst constructs are 31411.88 Da and 31633.18 Da, respectively. Characterization of the purified proteins by de-convoluted electrospray mass spectrometry showed their molecular weights to be in agreement with their theoretical masses to <0.58Da (Pth at 31.4 kDa and Gst at 31.6 kDa). A correction was performed using an external standard (myoglobin).

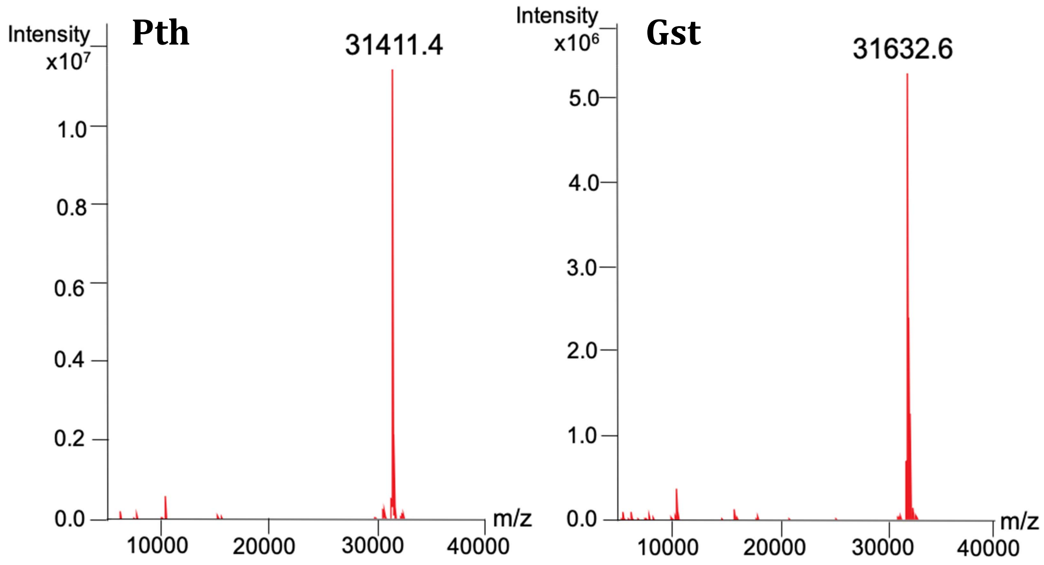

Figure S2 Characterization of the expressed constructs of the Pth and Gst proteins using mass spectrometry. The electrospray mass spectra show two distinct peaks at high intensities corresponding to the theoretical masses of Pth 31.4 kDa (left panel) and Gst at 31.6 kDa (right panel).

Correct folding of the proteins was confirmed using CD spectroscopy performed on a Jasco J810 CD spectropolarimeter at 20 °C under constant nitrogen flush, with the spectra recorded on a Jasco DIP-370 digital polarimeter. Experiments were carried out at 20 °C using a 2 mm pathlength quartz cell. After dialysis against MilliQ water, the protein solutions were diluted with MilliQ water to produce

concentrations of ~0.25 mg/ml. Random error and noise were reduced for each spectrum by averaging five scans in the wavelength range 260–185 nm. The signal from MilliQ water was subtracted from the protein spectra using the Jasco software. The two thermophilic proteins are seen to be correctly folded with clearly identifiable secondary structure composition.

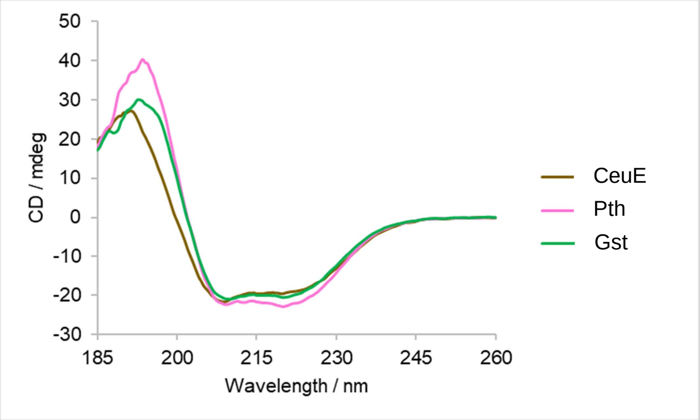

Figure S3 Circular dichroism spectra of CjCeuE, Gst and Pth proteins. All are seen to be folded with the expected bands for helices and sheets.

```
Mature CjCeuE
  1 CNSNSNENNA SSTTKTNTAT VKVLPISMSD EGDSFLVKDS LGENKIPKNP SKVVILDLGI LDTFDALKLN DKVVGVPAKN
 81 LPKYLQQFKN KPSVGGVQQV DFEAINALKP DLIIISGRQS KFYDKLKEIA PTLFVGLDNA NFLSSFENNV LSVAKLYGLE
161 KEALEKISDI KNEIEKAKSI VDEDKKALII LTNSNKISAF GPQSRFGIIH DVLGINAVDE NIKVGTHGKS INSEFILEKN
241 PDYIFVVDRN VILGNKERAQ GILDNALVAK TKAAQNKKII YLDPEYWYLA SGNGLESLKT MILEIKNAVK
Mature Gst
  1 CGNKENASNG ASGKNDEKKT EQAEEMTIKH QLGEAKVKKN PEKVVVFDFG VLDTLDKLGV KVTALPQMNV PKYLEKYKSS
 81 DYQNVGSLME PDFEKLSEIK PDVIFISGRQ ANLYDKLKEI GPTVYIGIDT QHYWDSFTNN MKLIGQMFGK EKEVDEELAN
161 IEKQIEEVKT KAADKKALII LTTGGKVSAY GKGSRFGLIH DVLGVPAADP NLKVTNPHGQ SVSFEYIAEK NPDYLFVIDR
241 DAVVEGKPTA KQTIENALVK KTKAYQNGHI VYLDPNYWYL SGGGLTSVSE MIKQVEEGLK
Mature Pth
  1 CGNEDNAKNA SSSNPKNDSE EITIKHELGE TKVKKKPEKV VVFDFGVLDS LDKLGVEVTG VPKANLPSYL EKYKDSKYEN
 81 VGGLMEPDFE KINEIAPDLI IISGRQANSY EKFAEIAPTV YMGIDTKNYI DSFANNMKTL GKIFGKEKEV EKELESINKQ
161 IEAVKAKAEK TSGKALIVLT TGGKVSAYGP GSRFGIIHDV LGIKPVDANI EVSTHGQSIS FEYIAEKNPD YLFVVDRDAV
241 VAGKPSAKQT IENELVKKTN AYKNNRIIYL NPNYWYLAGG GLISVAEMIN EVEKGIE
```

Figure S4 The sequences of the three individual mature proteins starting from the N-terminal cysteines. The regions shown in red were excluded from the constructs used herein.

A

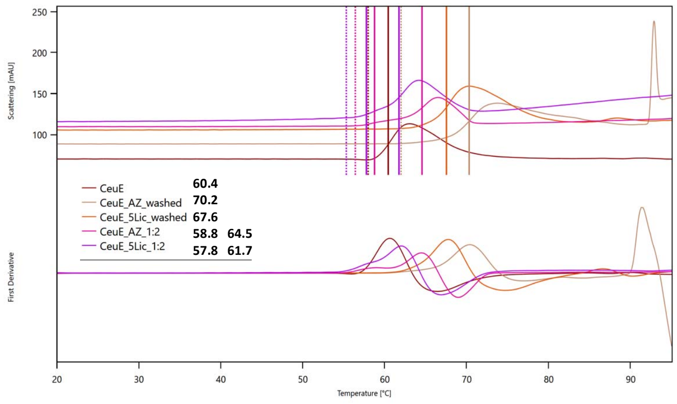

B

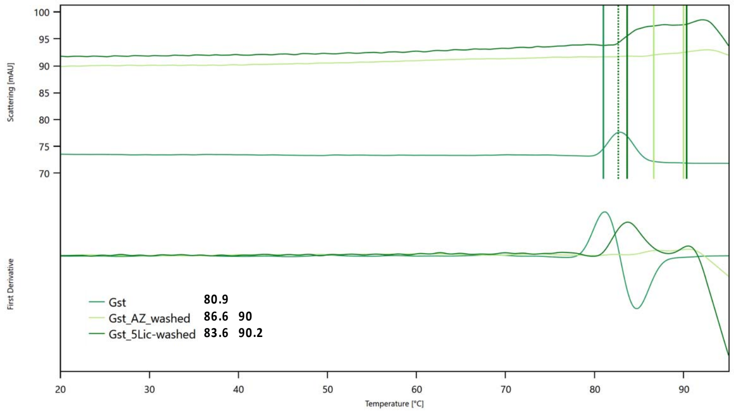

C

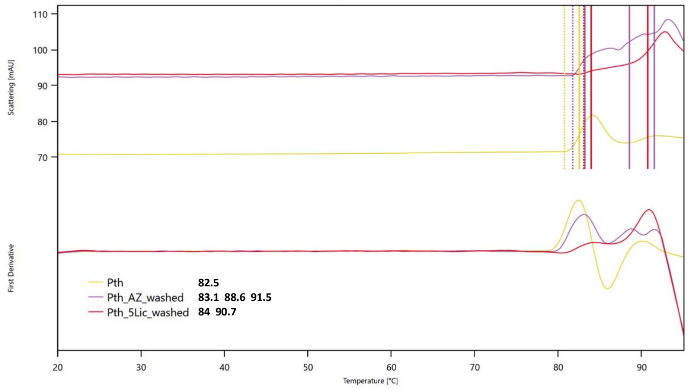

Figure S5 Thermostability assays of *Cj*CeuE (**A**), Gst (**B**) and Pth (**C**) unliganded and complexed with both Fe^{III}-azotochelin and Fe^{III}-5-LICAM. Unfolding curves only were recorded from 20-95 °C. First derivatives are plotted from the F350/330 ratios. The vertical lines represent the highest point of each peak, i.e. the T_m values. The apo Pth (yellow) and Gst (green) proteins have higher T_m values and are substantially more thermostable than *Cj*CeuE (purple). The Pth T_m is 82.5 °C, Gst T_m is 80.9 °C and the *Cj*CeuE T_m is 60.4 °C. The refolding phase shows no points of inflection: once the proteins have been denatured, they do not refold into their native forms when the temperature is decreased back to 20 °C. The T_m values increased on ligand binding for all three proteins.

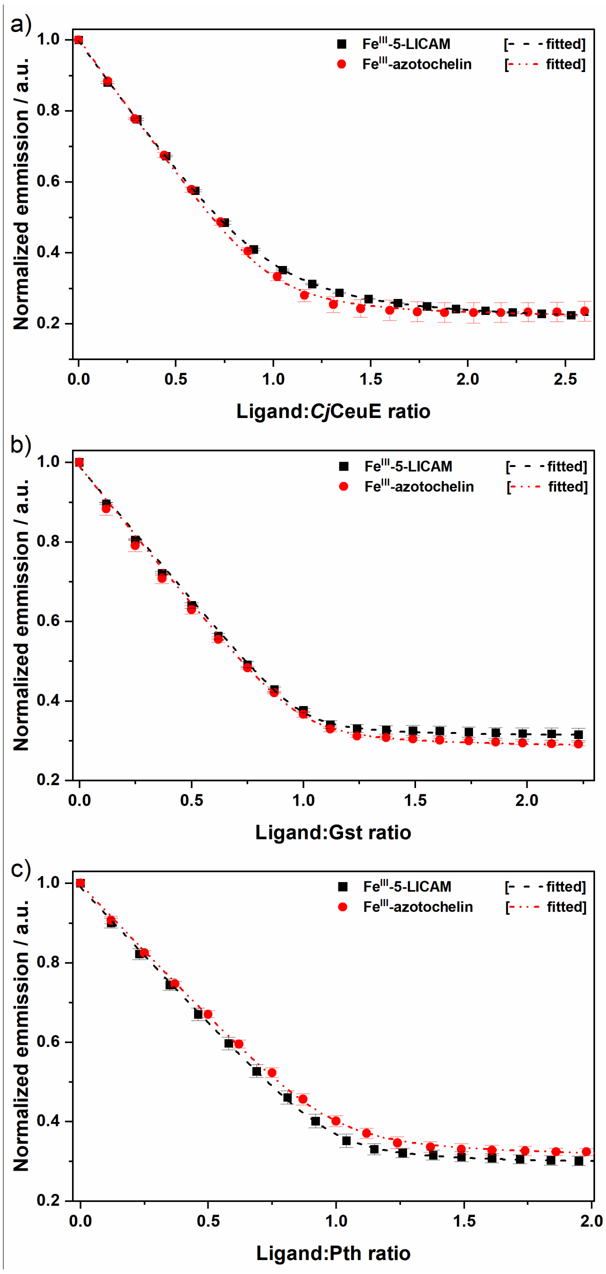

Figure S6 Fluorescent quenching observed on addition of Fe^{III}-5-LICAM and Fe^{III}-azotochelin to *Cj*CeuE (a), Gst (b) and Pth (c). Stock solution concentrations as follows: [*Cj*CeuE] & [Fe^{III}-5-LICAM] = 300 nM & 90 µM. [*Cj*CeuE] & [Fe^{III}-azotochelin] = 240 nM & 70 µM. [Gst] & [Fe^{III}-5-LICAM] = 180 nM & 55.5 µM. [Gst] & [Fe^{III}-azotochelin] = 240 nM & 60 µM. [Pth] & [Fe^{III}-5-LICAM] = 240 nM & 45 µM. [Pth] & [Fe^{III}-azotochelin] = 240 nM & 60 µM.

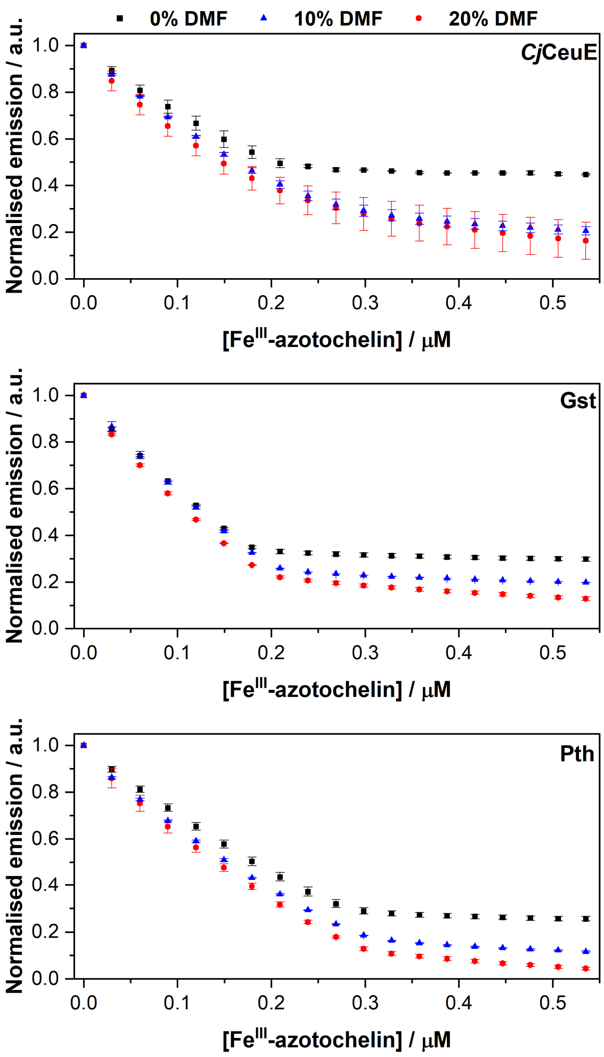

Figure S7 Fluorescent quenching observed on addition of Fe^{III}-azotochelin to *Cj*CeuE, Gst and Pth under varied buffer:DMF ratios (e 20% DMF). Protein stock solutions in buffer:DMF and ligand concentrations are as follows: [*Cj*CeuE] & [Fe^{III}-azotochelin] = 257 nM & 60 μM. [Gst] & [Fe^{III}-azotochelin] = 191 nM & 60 μM. [Pth] & [Fe^{III}-azotochelin] = 250 nM & 60 μM. Buffer: 40 mM Tris-HCl, pH 7.5, 150 mM NaCl. Average data points are shown with respective mean absolute deviations. K_d values (10% / 20% DMF): CjCeuE (11.3 ± 3.3 /15.3 ± 4.5), Gst (3.7 ± 0.4 / 4.8 ± 1.2), Pth (14.6 ± 2.4 / 15.1 ± 2.3).

S1. Experimental extinction coefficients for *Cj*CeuE, Pth and Gst

The extinction coefficients ($\boldsymbol{\varepsilon_E}$) of *Cj*CeuE, Pth and Gst were determined according to a protocol previously described (Harms, 2013). The procedure detailed below corrects the theoretical extinction coefficient ($\boldsymbol{\varepsilon_T}$), which only considers single residue contributions in the calculations, as if the protein is fully denatured, and does not consider any contribution from the folded protein. We experimentally measured the ratio between the absorbance at 280 nm for the folded protein in Tris/HCl buffer ($\boldsymbol{A_{0M}}$)

and fully denatured protein in the same buffer containing 6 M guanidine hydrochloride (GndHCl) (A_{6M}), and determined the corrected extinction coefficient (ε_E) according to equation below:

$$\varepsilon_E = \frac{A_{0M}}{A_{6M}}\,\varepsilon_T$$

The theoretical extinction coefficients (ε_T) were calculated using ProtParam (https://web.expasy.org/cgi-bin/protparam/protparam) by entering the sequences below:

***Cj*CeuE:**

GPAMLPISMSDEGDSFLVKDSLGENKIPKNPSKVVILDLGILDTFDALKLNDKVVGVPAKNLPKYLQQ
FKNKPSVGGVQQVDFEAINALKPDLIIISGRQSKFYDKLKEIAPTLFVGLDNANFLSSFENNVLSVAK
LYGLEKEALEKISDIKNEIEKAKSIVDEDKKALIILTNSNKISAFGPQSRFGIIHDVLGINAVDENIK
VGTHGKSINSEFILEKNPDYIFVVDRNVILGNKERAQGILDNALVAKTKAAQNKKIIYLDPEYWYLAS
GNGLESLKTMILEIKNAVK

Gst:_

GPATEQAEEMTIKHQLGEAKVKKNPEKVVVFDFGVLDTLDKLGVKVTALPQMNVPKYLEKYKSSDYQN
VGSLMEPDFEKLSEIKPDVIFISGRQANLYDKLKEIGPTVYIGIDTQHYWDSFTNNMKLIGQMFGKEK
EVDEELANIEKQIEEVKTKAADKKALIILTTGGKVSAYGKGSRFGLIHDVLGVPAADPNLKVTNPHGQ
SVSFEYIAEKNPDYLFVIDRDAVVEGKPTAKQTIENALVKKTKAYQNGHIVYLDPNYWYLSGGGLTSV
SEMIKQVEEGLK

Pth:_

GPAKNDSEEITIKHELGETKVKKKPEKVVVFDFGVLDSLDKLGVEVTGVPKANLPSYLEKYKDSKYEN
VGGLMEPDFEKINEIAPDLIIISGRQANSYEKFAEIAPTVYMGIDTKNYIDSFANNMKTLGKIFGKEK
EVEKELESINKQIEAVKAKAEKTSGKALIVLTTGGKVSAYGPGSRFGIIHDVLGIKPVDANIEVSTHG
QSISFEYIAEKNPDYLFVVDRDAVVAGKPSAKQTIENELVKKTNAYKNNRIIYLNPNYWYLAGGGLIS
VAEMINEVEKGIE

Experimental procedure: buffer A (0.05 M Tris-HCl / 0.15 M NaCl / pH 7.5) and buffer B (0.05 M Tris-HCl / 0.15 M NaCl / 6 M GndHCl / pH 7.5) were prepared using HPLC grade water and filtered through 0.45 µm nylon syringe filters before use. Before use, quartz cuvettes (1 cm path length) were soaked in 1 M HCl, rinsed several times with distilled water, and then dried under N_2 flow. Stocks of concentrated proteins were prepared in buffer A (absorbance at 280 nm > 1). 200 µL of protein stock was then diluted in 1800 µL buffer and left resting for 10 minutes before recording the UV-vis spectra at medium speed from 500 to 250 nm. Triplicates for each buffer (A or B) were recorded for each protein (Figure S8), and data presented at Table S1.

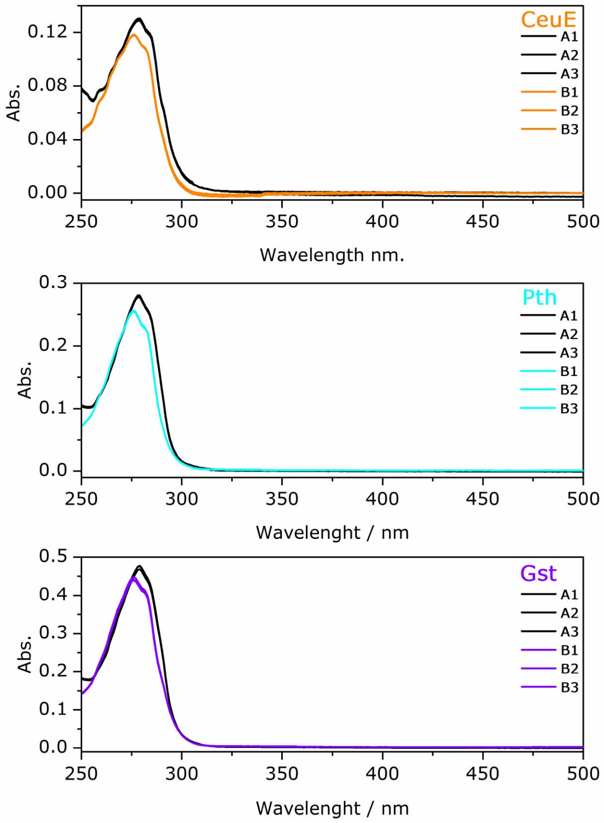

Figure S8 UV-vis spectra of *Cj*CeuE, Gst and Pth in Buffer A and B, recorded in triplicate for each protein/buffer pair. Average absorbance at 280 nm at 0 or 6 M guanidine hydrochloride (Table S1) were used to determine the respective experimental molar extinction coefficients for *Cj*CeuE, Gst and Pth.

Table S1 Crystals of Gst and Pth used for structure determination.

Protein and Ligand	Crystallisation conditions
**Gst apo**	Index D3: 0.1 M HEPES pH 7.0, 30% Jeffamine ED 2001, pH 7.0. No cryo added.
**Gst** Fe^{III}**-azotochelin**	Morpheus C8: 12.5% PEG1K; 12.5% PEG 3350; 12.5% MPD; 0.03 M each NPS ($NaNO_3$, Na_2HPO_4, $(NH_4)_2SO_4$); 0.1 M MOPS/HEPES pH7.5. Ligand: 1.67 mM Fe azo-sulf/pyr-Ir catalyst, 1:1 protein:ligand ratio. No cryo added.
**Gst** Fe^{III}**-5-LICAM**	Morpheus A12: 12.5% PEG 1000; 12.5% PEG 3350; 12.5% MPD; 0.03 M $MgCl_2$, $CaCl_2$, 0.1 M Bicine/TRIS, pH 8.5. Ligand: 5mM Fe^{III} 5-LICAM 1:10 ratio protein:ligand. No cryo added.
**Pth apo**	Opt: 17% PEG 3350; 0.1 M MES, pH 6.5, 0.1 M MgAc; Cryo- Ethylene Glycol.
**Pth** Fe^{III}**-azotochelin**	Pact C2: 0.1 M PCB pH 4.0 (Na propionate: NaCacodylate: BisTRIS propane 2:1:2); 25% PEG 1.5K). Ligand: 5d mM Fe^{III} azotochelin (Washed) 1:10 ratio protein:ligand. No cryo added.
**Pth** Fe^{III}**-5-LICAM**	Opt: 18% PEG 3350, 0.1M TRIS, pH 8.5, 0.05M ZnAc. Cryo – ethylene glycol. Ligand: 5mM Fe^{III} 5-LICAM 1:10 ratio protein:ligand.

Table S2 Experimental molar extinction coefficients determination for *Cj*CeuE, Gst and Pth.

Scaffold	e	A_{6M}	ε_T	$\varepsilon_E = \frac{A_{0M}}{A_{6M}} \varepsilon_T$
***Cj*CeuE**	0.1288 ± 0.0002	0.1104 ± 0.0006	15930 M^{-1} cm^{-1}	**18585 M^{-1} cm^{-1}**
**Gst**	0.469 ± 0.005	0.416 ± 0.004	30370 M^{-1} cm^{-1}	**34239 M^{-1} cm^{-1}**
**Pth**	0.275 ± 0.02	0.2340 ± 0.0009	24870 M^{-1} cm^{-1}	**29196 M^{-1} cm^{-1}**

Harms, M. (2013). *Measuring protein concentration by absorbance.* https://harmslab.uoregon.edu/wiki/harms-lab-protocols/protein-experiments/measuring-concentration-with-nanodrop/.
